# Exploring human hair degradation: A preliminary study for estimating time-since-death

**DOI:** 10.1007/s00414-025-03476-4

**Published:** 2025-03-26

**Authors:** Angela Silva-Bessa, Stuart Ramage, Maria Teresa Ferreira, Ricardo Jorge Dinis-Oliveira, Shari L. Forbes, Lorna Dawson

**Affiliations:** 1 Centre for Functional Ecology, Laboratory of Forensic Anthropology, Department of Life Sciences, University of Coimbra, Coimbra, Portugal; 2 Associate Laboratory i4HB - Institute for Health and Bioeconomy, University Institute of Health Sciences – CESPU, 4585-116 Gandra, Portugal; 3 UCIBIO - Applied Molecular Biosciences Unit, Forensics and Biomedical Sciences Research Laboratory, University Institute of Health Sciences (1H-TOXRUN, IUCS-CESPU), 4585-116 Gandra, Portugal; 4 School of Pharmacy and Life Sciences, Robert Gordon University, Aberdeen, Scotland; 5 Centre for Forensic Soil Science, Environmental and Biochemical Sciences Department, The James Hutton Institute, Aberdeen, Scotland; 6 UCIBIO - Applied Molecular Biosciences Unit, Translational Toxicology Research Laboratory, University Institute of Health Sciences (1H-TOXRUN, IUCS-CESPU), 4585-116 Gandra, Portugal; 7 Department of Public Health and Forensic Sciences, and Medical Education, Faculty of Medicine, University of Porto, Porto, Portugal; 8 FOREN - Forensic Science Experts, Avenida Dr. Mário Moutinho, 33-A, 1400-136, Lisbon, Portugal; 9 Department of Chemistry and Biochemistry, University of Windsor, Windsor, ON Canada

**Keywords:** ATR-FTIR, Microtaphonomy, Postmortem interval, Proteolysis

## Abstract

Postmortem interval (PMI) estimation is a challenging task in forensic investigations. PMI assessment frequently requires the application of the currently available methods which can lead to unsatisfactory results due to the poor accuracy of time interval estimation. To address these concerns, the present study aimed to evaluate whether there is a correlation between human hair proteolysis and PMI. Scalp hair samples of three living donors and eleven individuals exhumed from different burial types from Portuguese cemeteries were analysed by ATR-FTIR (attenuated total reflectance – Fourier-transform infrared). Four band areas and three hair degradation indices were considered in the 2000–1000 cm^−1^ spectral region. When analysing the entire dataset (i.e., 126 infrared spectra) – and when separating and analysing the spectroscopic data by burial type – the ratio between amide II (∼1550 cm^−1^) and S = O and SO_3_ combined (∼1074 cm^−1^ and ∼1043 cm^−1^, respectively) suggests there is a correlation between hair proteolysis and PMI (*p* < 0.05). Nevertheless, it is recommended that a larger dataset is required to confirm the preliminary results obtained in this study and to explore how this correlation can be used to estimate PMI in forensic casework.

## Introduction

When dealing with human remains, estimating time-since-death – also known as postmortem interval (PMI) – is often one of the first steps involved in an investigation. Calculating how long an individual has been deceased is of the utmost importance for several main reasons: i) to determine the medico-legal relevance of the case; ii) to assist with victim identification and checking of alibis; and iii) to provide an accurate time window of when someone had gone missing. Despite its legal significance, estimating PMI with accuracy is still challenging, especially when it comes to human remains in the advanced stages of decomposition, burnt, mummified, or skeletonised [1, 2]. This is because the interactions between endogenous and exogenous variables are not straightforward, and the decomposition process is unlikely to act the same in all bodies in all circumstances [3, 4]. Furthermore, the existing methods have some limitations given that they can only be applied under specific circumstances, as is the case with entomology which can be useful in the presence of insects [5] or the decomposition chemistry of muscle proteins which can be applied when soft tissues are recovered [6, 7]. Thus, new approaches are needed to estimate the PMI of human remains in situations where other methods cannot be used.

**Fig. 1:**
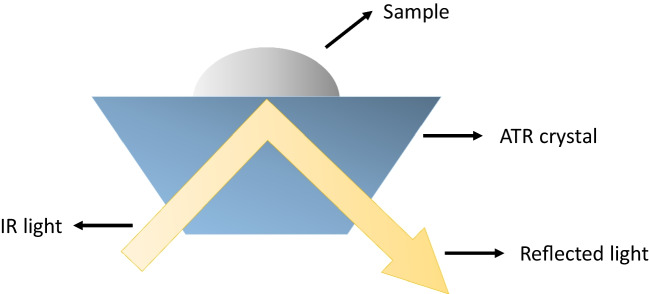
Schematic representation of an ATR-FTIR experiment

Attenuated total reflection (ATR) Fourier-transform infrared (FTIR) spectroscopy is a technique that has been gaining popularity in the analysis of biological samples for over a decade [1, 8, 9]. FTIR spectroscopy is a vibrational spectroscopic technique whose spectral bands are molecule specific and are able to provide information regarding the biochemical composition of the analysed sample [9]. The infrared (IR) spectral region can be divided into near-IR (13000–4000 cm^−1^), mid-IR (4000–200 cm^−1^) and far-IR (< 200 cm^−1^) [10]. Once the IR radiation interacts with the sample, the light can be reflected and, in reflectance mode, it will interact with the surface of the sample instead of passing through it. In ATR sampling, the IR light travels through a crystal, is reflected internally at the crystal-surface interface, and the reflected light travels to the FTIR detector (Fig. 1).

The hair shaft structure can be divided into three main layers: i) cuticle; ii) cortex; and iii) medulla. The cuticle is the outermost layer of a hair fibre, comprising up to ten overlapping keratinised cells that work as a shield against external damage [11, 12]. On the other hand, the cortex comprises of spindle-shaped cortical cells, each one of them comprising of macrofibrils which are, in turn, made up of smaller microfibrils (i.e., keratin intermediate filaments) [11, 12]. It is also the cortex that contains melanosomes. Melanosomes are membrane-bound cytoplasmic organelles unique to melanocytes that can synthesise melanin, being responsible for the wide spectrum of dark to light hair colour pigmentation [13, 14]. In human hair, the medulla varies in its appearance and size, sometimes being absent from hair strands. When present, the medulla is a spongy structure that can either be continuous or interrupted along the fibre, and is filled with air or fluid [11]. Furthermore, this hair layer comprises of medullary trichohyalin granules which are keratin-associated proteins (KAPs) only found in the hair shaft medulla [11].

Due to its inherent high content of keratin and KAPs, human hair can survive for millennia [11, 15]. Keratin is an insoluble fibrous protein whose rigid structure limits hair degradation and makes it a nutrient source only for specific keratinolytic microorganisms [16]. Keratin is rich in the amino acid cysteine which forms disulfide bonds that are key to the resilience and strength of hair strands [11]. Additionally, melanosomes also contain cysteine, whose presence is considered an important regulator of melanogenesis (i.e., melanin synthesis) [17, 18].

**Fig. 2:**
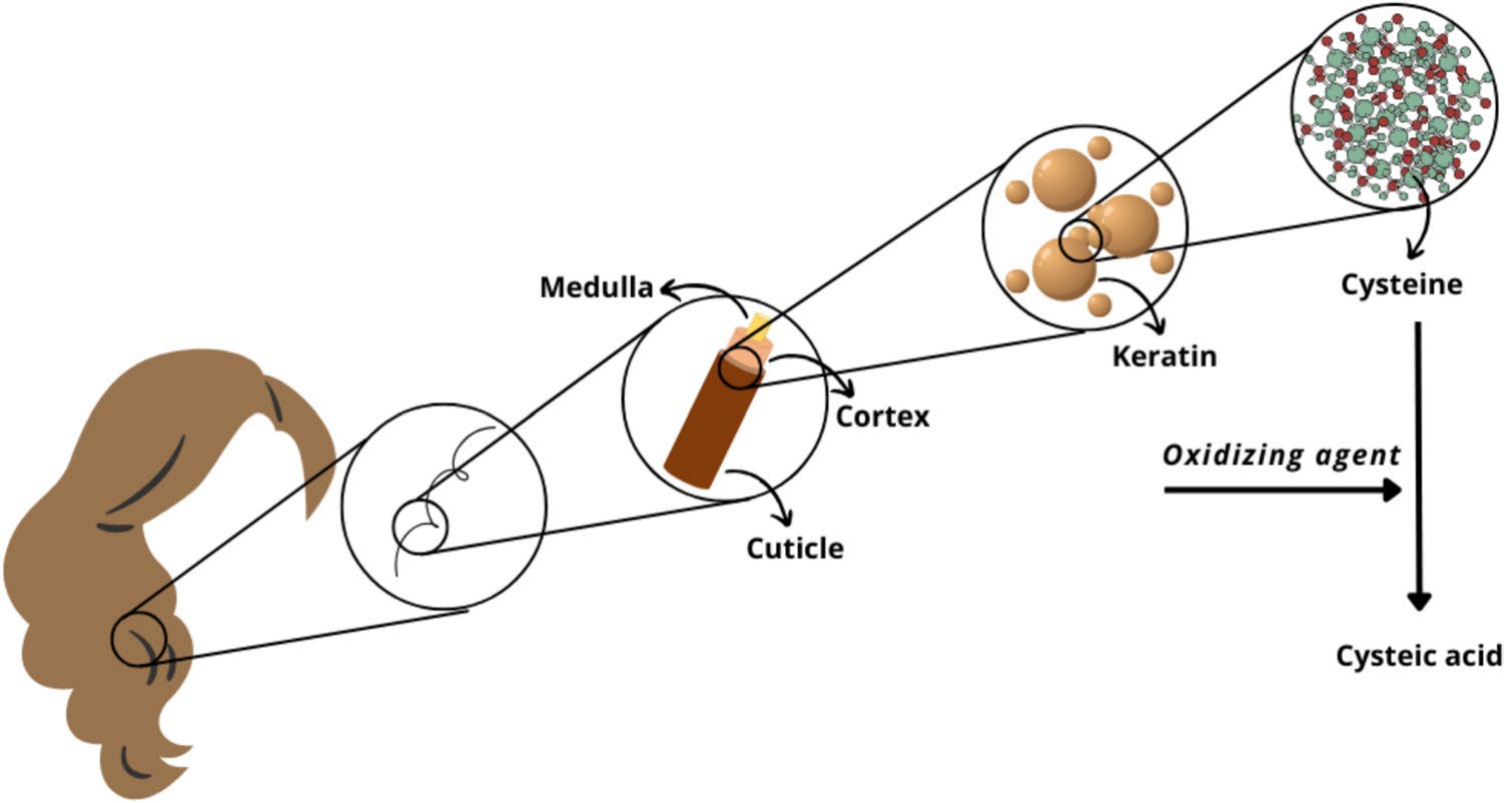
Schematic representation of the structure of a human scalp hair shaft and the oxidation of cysteine

In living individuals, hair disruption by bleaching products or natural sunlight makes space for the oxidation of cysteine to cysteic acid [19, 20] (Fig. 2). It is expected that taphonomic processes (i.e., alterations occurring after death) will likely have the same effect in the breakdown of cysteine, even though it might occur much slower and at a microscale [21, 22]. Changes in the structure of proteins can be detected in IR spectra, with the oxidation of cysteine to cysteic acid resulting in an increase of the disulfide monoxide (S = O) stretching band caused absorbance. For this reason, this preliminary study aimed to determine if cysteine oxidation could be used for PMI estimation. The ATR-FTIR spectroscopic technique was applied on scalp hair samples of eleven deceased individuals exhumed from Portuguese cemeteries and on scalp hair from three living donors with an emphasis on four band areas in the mid-IR spectral region, more specifically between 2000–1000 cm^−1^. Scanning electron microscopy (SEM) was also used to observe the physical degradation of the hair samples.

## Material and methods

### Research context

In Portugal, it is common practice to bury a deceased individual in an aerobic consumption module, a vault, or a soil grave which can either be temporary or perpetual [23]. In all these situations, coffins can only be opened after a minimum period of three years as established by the Portuguese Decree-Law No. 411/98 of 30 December 1998 [24]. If the remains still contain soft tissue (e.g., muscle and skin) at the time of opening, the inhumation must continue for successive periods of two years until complete skeletonisation is achieved. Once the body is completely skeletonised, bones, garments, and coffin materials are permitted to be removed. The skeletal remains can either then be cremated or transferred to an ossuary or family plot, and the burial place re-used for a new entombment.

### Burial conditions and examined bodies

A total of 11 burial openings took place with the permission of the relatives of the deceased and on behalf of the city halls involved in this study as established by law (Decree-Law No. 411/98 of 30 December 1998 [24]). Grave openings occurred in five Portuguese public cemeteries from four different cities: Braga (Cemetery of Monte d’Arcos – CMA), Porto (Cemetery of Prado do Repouso – CPR), Mértola (Cemetery of Encosta do Castelo – CCM), and Faro (Cemetery of Boa Esperança – CBE; Cemetery Novo de Faro – CNF).

Depending on the cemetery (Table 1), the individuals had been buried in aerobic consumption modules (n = 2; 18.2%), vaults (n = 4; 36.4%), or soil graves (n = 5; 45.5%). The examined bodies (five females and seven males) had an age-at-death between 75 and 99 years-old (mean = 87.6; SD = 6.58); however, there were no records on the age-at-death of three individuals (one adult and two non-adults). The lack of information in these cases is owed to the poor registration system cemeteries had at the end of the nineteenth century/beginning of the twentieth century, with no information being available either on the burial headstone or on the coffin’s plate. PMI ranged from 6 to 131 years (mean = 34.9; SD = 39.2).

**Table 1:** Information on the deceased individuals from which scalp hair samples were obtained (CBE: Cemetery of Boa Esperança; CCM: Cemetery of Encosta do Castelo; CMA: Cemetery of Monte d’Arcos; CNF: Cemetery Novo de Faro; CPR: Cemetery of Prado do Repouso; PMI: postmortem interval; SEM: scanning electron microscope)

Individual ID	Burial type	Sex	Age-at-death (years)	PMI (years)	State of decay	SEM analysis
CBE/01	Soil	Male	92	7	Skeletonised	No
CBE/06	Soil	Female	99	6	Skeletonised	No
CCM/03	Soil	Male	84	28	Skeletonised	Yes
CMA/03	Soil	Male	84	6	Partially skeletonised & in putrefaction	Yes
CMA/19	Vault	Male	Non-adult*	40	Skeletonised	Yes
CMA/40	Vault	Female	87	40	Skeletonised	Yes
CNF/03	Aerobic consumption module	Male	90	10	Mummified	No
CNF/04	Aerobic consumption module	Male	90	16	Mummified	No
CPR/18	Vault	Female	Non-adult*	93	In putrefaction	Yes
CPR/26	Vault	Female	Adult	131	Skeletonised	Yes
CPR/48	Soil	Female	75	7	Partially skeletonised & mummified	No

^*^Individuals are considered non-adults when their age-at-death is below 21-years-old

Of the total number of burial openings and examined individuals (n = 11), six (54.5%) were completely skeletonised while five (45.5%) still contained desiccated or in putrefaction soft tissues (Table 1).

### Sample collection and handling

Scalp hair samples from all individuals (n = 11) were collected and stored following published guidelines [25, 26]. The quantity and length of the strands of hair collected varied according to what was available from each individual. Still, between two and seven strands of hair were attained. Hair was usually loose due to the degradation of cranial soft tissues, making it challenging to obtain the same shaft section for all individuals. No follicles were detected and the individuals’ hair shaft colouring varied between white, light blond, dark blond, and dark brown.

### Control samples

Three strands of hair were also obtained from a total of three living individuals (two females and one male) and stored following published guidelines [25, 26]. As illustrated in Table 2, the age of the living donors ranged between 26 and 60 years (mean = 38.3; SD = 15.4). The hair samples from the three living donors (LDS) were collected to act as control samples (i.e., PMI = 0). The strands of hair were collected with the follicle and varied between light blond and dark brown in colour.

**Table 2:** Information on the living donor individuals from which scalp hair samples were obtained (SEM: scanning electron microscope)

Individual ID	Sex	Age (years)	SEM analysis
LDS/01	Female	60	No
LDS/02	Female	29	No
LDS/03	Male	26	Yes

### Sample preparation for analysis

All hair samples were washed sequentially with Triton X-100 (0.5% v/v; Sigma-Aldrich, USA), ultrapure water and acetone (LabChem; Zelienople, PA, USA) to remove potential exogenous contamination, and air-dried overnight [4].

For ATR-FTIR analysis, a total of three strands of hair per individual were used for the analysis. A 1 cm section of each strand of hair was incised and mounted on a sticky carbon stub. Given that only two strands of hair were available for individuals CBE/01 and CMA/03, the longest strands of hair were split in half to produce a total of three sections of 1 cm length on the carbon stubs.

The same procedure was followed for SEM imaging of the hair samples for a total of seven individuals (Tables 1 and 2).

### ATR-FTIR spectroscopic analysis

Hair samples were subjected to ATR-FTIR analysis using a Nicolet™ iN10 FTIR microscope with a Ge tip ATR attachment. Along each of the 1 cm sections, three equidistant measurements were taken to evaluate intra- and inter-variation between the strands of hair of each individual. As such, a total of nine measurements per individual were obtained.

Data points were recorded over 128 scans using a liquid nitrogen-cooled MCT detector in the 4000–650 cm^−1^ range with a resolution of 4 cm^−1^. Air blank spectra were recorded before each measurement using the same number of scans and resolution. Spectra are displayed in absorbance.

Four IR band areas were obtained using the OMNIC software. The band areas recorded were: i) ∼1650 cm^−1^ (amide I); ii) ∼1550 cm^−1^ (amide II); iii) ∼1230 cm^−1^ and ∼1174 cm^−1^ combined (amide III (disordered) including the shouldering cysteic acid SO_3_ asymmetric stretch band, respectively); and iv) ∼1074 cm^−1^ and ∼1043 cm^−1^ combined (disulfide monoxide symmetrical stretch and shouldering SO_3_ symmetric stretch bands of cysteic acid, respectively). The selection of the aforementioned band areas was based on the publication of Santos and collaborators [20] whose research showed that the amide I and amide II bands decrease in intensity after the application of cosmetic products for hair bleaching while the concentration of cysteic acid increases.

### SEM imaging

Hair samples were subjected to variable pressure (VP) scanning electron microscopy (SEM) imaging using a Zeiss EVO LS10. Control of the microscope was carried out using the Zeiss Smart SEM software. This technique was applied to visualise topographic features of the hair strands such as external damage caused to the hair cuticle region. Stubs were coated with 2–5 nm of gold–palladium, and images were obtained with a magnification of 1000x and 3000x. The accelerating potential was 25 kV.

### Data analysis

**Fig.3:**
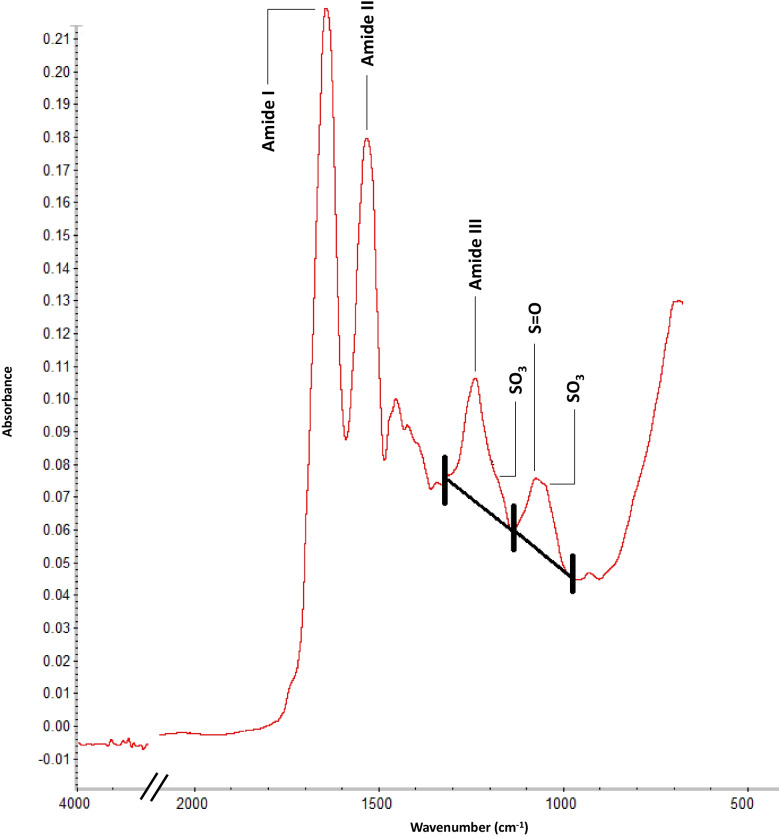
ATR-FTIR spectrum showing the four band areas considered for hair degradation indices (obtained from individual LDS/03). As expected from non-treated hair, the intensity of amide I and amide II bands are higher than the intensity of the two bands where cysteic acid is detected

Statistical analysis of the spectroscopic data was performed with R software (Version 4.2.2, R Foundation for Statistical Computing, Vienna, Austria). Initially, a descriptive statistic was performed for the band areas measured for all individuals included in this study. Afterwards, three ratios between specific IR band areas were calculated to determine which degradation index was better correlated with PMI (Fig. 3; Table 3). Data from living donors were included in all calculations with an implicit PMI of 0. The Spearman’s correlation coefficient (r_s_) was then used to assess the relationship between the degradation indices and PMI. Spearman's correlation determines the strength and direction of the monotonic relationship between two variables. This means that two variables might converge but not at a constant rate. A Spearman’s correlation coefficient can be weak (from 0 to 0.3), moderate (from 0.4 to 0.6), or strong (from 0.7 to 1) in both positive and negative correlations. Lastly, the spectroscopic data was divided according to the burial type and correlations were assessed considering this variable. The level of significance was set at *p* < 0.05. No outliers were removed from the database given that it would be counterproductive and potentially introduce bias to this preliminary study.

**Table 3:** Degradation indices considered based on the ratios of band areas and respective spectral relationships

	Ratio	Spectral relationship
Degradation index I	(Amide I) / (S = 0 + SO_3_)	Abs(∼1650 cm^−1^) / Abs(∼1074 cm^−1^ + ∼1043 cm^−1^)
Degradation index II	(Amide II) / (S = 0 + SO_3_)	Abs(∼1550 cm^−1^) / Abs(∼1074 cm^−1^ + ∼1043 cm^−1^)
Degradation index III	(Amide III + SO_3_) / (S = 0 + SO_3_)	Abs(∼1230 cm^−1^ + ∼1174 cm^−1^) / Abs(∼1074 cm^−1^ + ∼1043 cm^−1^)

## Results

**Fig. 4:**
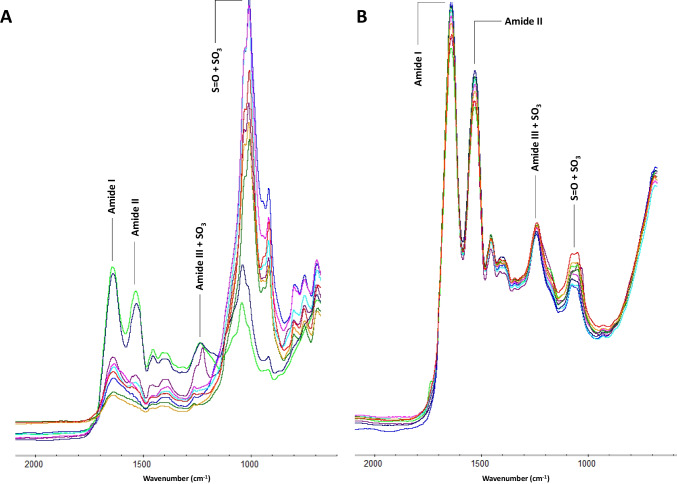
ATR-FTIR spectra of individuals CPR/26 (PMI = 131 years) (**A**) and CCM/03 (PMI = 28 years) (**B**) showing the four band areas considered for hair degradation indices

A total of 126 IR spectra were attained, showing intra- and inter-variability in the hair samples analysed (Fig. 4). Table 4 summarises the actual values obtained, according to each individual and the four band areas under study. As demonstrated by the calculated standard deviation (SD), the combination of amide III and the shouldering cysteic acid SO_3_ asymmetric stretch band showed the lowest spread of the values in the dataset. To understand if the SD values achieved were in fact low, the coefficient of variation (CoV) was assessed. In doing so, it would be possible to check the stability of amide I and amide II, and ensure they were appropriate bands to use to compare changes against the cysteic acid bands. Here, the amide I band showed the lowest CoV (i.e., the lowest SD relative to the mean).

**Table 4:** Descriptive statistics for the band areas considered for hair degradation according to each individual under study (CBE: Cemetery of Boa Esperança; CCM: Cemetery of Encosta do Castelo; CMA: Cemetery of Monte d’Arcos; CNF: Cemetery Novo de Faro; CPR: Cemetery of Prado do Repouso; CTR: control; SD: standard deviation; CoV: coefficient of variation)

	**Amide I**		**Amide II**		**Amide III + SO**_**3**_		**S = 0 + SO**_**3**_	
	*Range* *(mean* ± *SD)*	*CoV*	*Range* *(mean* ± *SD)*	*CoV*	*Range* *(mean* ± *SD)*	*CoV*	*Range* *(mean* ± *SD)*	*CoV*
**CBE/01**	8.05 – 11.99 (10.36 ± 1.36)	0.13	4.07 - 6.31 (5.24 ± 0.74)	0.14	1.61 - 2.79 (2.41 ± 0.41)	0.17	1.54 - 10.83 (3.56 ± 3.17)	0.89
**CBE/06**	10.31 – 12.33 (11.68 ± 0.64)	0.05	5.36 - 6.39 (5.93 ± 0.35)	0.06	2.39 - 3.85 (2.99 ± 0.50)	0.17	1.40 - 1.86 (1.66 ± 0.17)	0.10
**CCM/03**	9.84 – 11.78 (10.83 ± 0.60)	0.06	4.98 - 6.17 (5.60 ± 0.41)	0.07	2.76 - 3.44 (2.96 ± 0.21)	0.07	1.58 - 2.55 (2.05 ± 0.36)	0.17
**CMA/03**	3.18 – 10.41 (6.86 ± 2.72)	0.40	1.26 - 5.36 (3.18 ± 1.60)	0.50	0.88 - 2.22 (1.53 ± 0.44)	0.29	3.40 - 24.15 (12.11 ± 7.39)	0.61
**CMA/19**	4.65 – 11.95 (9.48 ± 2.11)	0.22	1.87 - 5.89 (4.73 ± 1.24)	0.26	1.85 - 3.59 (2.67 ± 0.58)	0.22	1.67 - 5.86 (2.68 ± 1.30)	0.49
**CMA/40**	4.88 – 9.45 (7.31 ± 1.76)	0.24	1.51 - 4.08 (2.75 ± 1.13)	0.41	0.51 - 1.30 (0.94 ± 0.28)	0.30	2.61 - 18.69 (9.98 ± 6.57)	0.66
**CNF/03**	7.40 – 10.79 (9.77 ± 1.21)	0.12	3.80 - 5.50 (4.94 ± 0.63)	0.13	1.53 - 3.06 (2.43 ± 0.52)	0.21	1.36 - 2.21 (1.88 ± 0.25)	0.13
**CNF/04**	8.52 – 12.10 (11.07 ± 1.14)	0.10	4.54 - 6.09 (5.47 ± 0.55)	0.10	2.22 - 3.81 (3.10 ± 0.53)	0.17	1.47 - 2.10 (1.70 ± 0.22)	0.13
**CPR/18**	10.54 – 11.69 (11.14 ± 0.43)	0.04	5.26 - 5.88 (5.63 ± 0.26)	0.05	2.11 - 3.38 (2.82 ± 0.37)	0.13	1.41 - 1.95 (1.55 ± 0.16)	0.10
**CPR/26**	1.99 – 7.60 (4.25 ± 2.24)	0.53	0.33 - 3.50 (1.51 ± 1.16)	0.77	0.02 - 2.21 (0.73 ± 0.94)	1.29	6.18 - 48.96 (32.25 ± 14.68)	0.46
**CPR/48**	9.65 – 12.09 (11.06 ± 0.80)	0.07	4.86 - 6.30 (5.63 ± 0.48)	0.09	2.63 - 4.11 (3.52 ± 0.42)	0.12	1.47 - 2.38 (1.79 ± 0.28)	0.16
**CTR/01**	11.43 – 12.16 (11.83 ± 0.27)	0.02	6.11 - 6.37 (6.21 ± 0.08)	0.01	2.13 - 2.97 (2.56 ± 0.27)	0.05	1.52 - 1.99 (1.69 ± 0.15)	0.09
**CTR/02**	9.34 – 11.67 (10.54 ± 0.71)	0.07	5.00 - 5.81 (5.46 ± 0.27)	0.05	2.84 - 3.29 (2.97 ± 0.15)	0.05	1.40 - 1.60 (1.48 ± 0.08)	0.05
**CTR/03**	7.48 – 9.31 (8.34 ± 0.65)	0.08	5.64 - 6.03 (5.79 ± 0.13)	0.02	2.36 - 2.80 (2.57 ± 0.13)	0.05	0.95 - 1.73 (1.55 ± 0.30)	0.19

The Spearman’s correlation coefficients between PMI and the degradation indices are summarised in Table 5. Firstly, tests were conducted across the fourteen individuals and statistically significant differences were found for all degradation indices (*p* < 0.05). Despite the negative relationships obtained, these were only considered weak or moderate (index I: r_s_ = −0.35; index II: r_s_ = −0.45; index III: r_s_ = −0.35). When dividing the spectroscopic data according to the burial type, results came out differently. No statistically significant differences were found for the degradation index I on aerobic consumption modules (*p* = 0.37), nor on degradation index III for aerobic consumption modules (*p* = 0.47) and soil graves (*p* = 0.11). The obtained data showed that the degradation index II seems to be the one that fits better for PMI estimation in all burial types. Here, *p*-values were 8.42e^−4^ in aerobic consumption modules, 3.4e^−4^ in soil graves, and 1.14e^−6^ in vaults. The Spearman’s correlation coefficient also evidenced a moderate negative relationship in all situations with r_s_ values of −0.48 for aerobic consumption modules, −0.41 for soil graves, and −0.57 for vaults.

**Table 5:** Results obtained for Spearman’s correlation coefficients (r_s_) between the three degradation indices under study and PMI. Values in brackets include the number of samples from control individuals (n = 3) which were added to each situation for PMI = 0

	***n***	***PMI*** *(mean* ± *SD****)***	**Degradation index I**		**Degradation index II**		**Degradation index III**	
			*p*-value	r_s_	*p*-value	r_s_	*p*-value	r_s_
**All individuals**	11 (14)	34.9 ± 39.2	6.19e^−5^	−0.35	1.44e^−7^	−0.45	4.68e^−5^	−0.35
**Burial type**								
*Aerobic consumption modules*	2 (5)	13 ± 3	0.37	−0.14	8.42e^−4^	−0.48	0.47	−0.11
*Soil graves*	5 (8)	10.8 ± 8.61	0.02	−0.27	3.4e^−4^	−0.41	0.11	−0.19
*Vaults*	4 (7)	76 ± 38.4	4.61e^−5^	−0.49	1.14e^−6^	−0.57	1.34e^−5^	−0.52

**Fig. 5:**
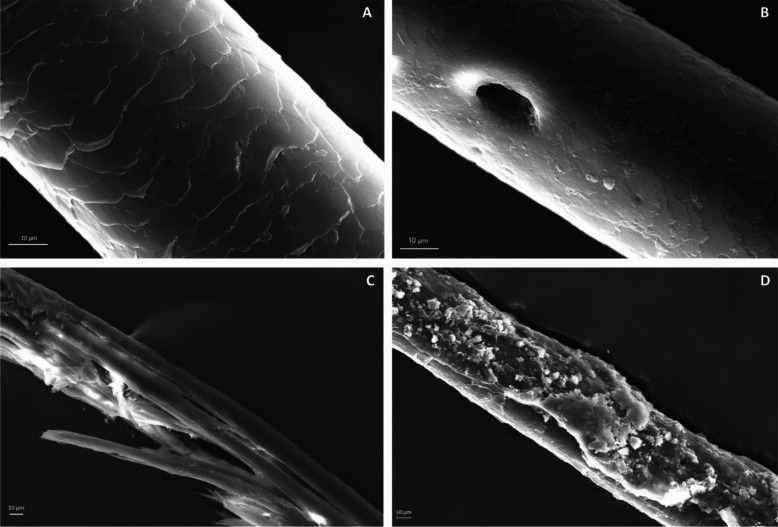
Scanning electron microscope (SEM) images acquired (3000x) of a hair strand shaft from individual LDS/03 showing the variability of its keratinised cells (**A**) and from individual CMA/19 (PMI = 40 years) showing what appears to be a fungal tunnel (**B**). SEM image acquired (1000x) from individual CMA/40 (PMI = 40 years) illustrating hair split (**C**) and from individual CMA/03 (PMI = 6 years) with the presence of mycelial masses (**D**)

As illustrated in Fig. 5, SEM images confirmed the physical degradation of three of the samples observed (CMA/19, CMA/40 and CPR/18). The presence of tunnels in the hair shaft of individual CMA/19 indicates that this alteration was mediated by fungi [15] with no other hair samples showing the same type of damage. Additionally, it was possible to observe hair split on individual CMA/40 and loss of the cuticle layer on individual CPR/18. Except for LDS/03, all hair samples also exhibited what appeared to be mycelial masses.

## Discussion

Changes in human hair recovered from different burial environments have been studied, and the oxidation of proteins has been used to evaluate the extent of degradation in long-buried hair samples [22, 27, 28]. The degradation indices considered in this study (Table 3) come from the change in ratio of cysteic acid peaks. The spectral region 1174–1040 cm^−1^ is assigned to cysteic acid and its intermediate oxides [20]. Here, the asymmetrical stretching of cysteic acid is detected at ∼1174 cm^−1^ while the symmetrical stretching of cysteic acid is observed at ∼1043 cm^−1^. In living individuals, the content of cysteic acid in hair increases through photochemical oxidation after exposure to natural radiation or chemical procedures such as bleaching or dyeing [20], and its formation is mostly evidenced by the increase in intensity of the band at ∼1043 cm^−1^ because the oxidation of the disulfide bonds (S–S to S = O) forms cysteic acid residues [29]. As illustrated in Table 4, this also occurred with the samples under study: overall, the band areas that combine both the disulfide monoxide symmetrical stretch (S = O) and the shouldering SO_3_ symmetric stretch of cysteic acid have the highest SD relative to the mean, indicating the biggest change over time.

According to the literature, the spectral profile of amides I, II, and III also suffer some changes due to the action of external agents [11, 20, 29, 30]. The degree of spectral variation of the amide bands appears to be related with the type of hair treatment (e.g., bleach or shikimic acid) [20, 30, 31] which also seems to react differently in hair samples from individuals with distinct biological affinities (e.g., European or African) [29]. It is important to note that no antemortem information was available regarding the natural hair pigmentation of the deceased individuals nor on any hair treatment conducted during life. However, it is known that melanocytes produce two types of melanin: yellow pheomelanin and black eumelanin [13]. The production of yellow to reddish-brown pheomelanin occurs when melanosomes contain high contents of cysteine [18]. Even though a negative correlation is always expected between the levels of cysteine and cysteic acid through time, hair pigmentation can inherently influence the content of cysteic acid after oxidation and it should be considered for future studies. Furthermore, although the deceased individuals are of Portuguese nationality, ancestry has not been confirmed either by antemortem data nor by postmortem analysis. For this reason, and because sex and age-at-death are also not known for all individuals, the biological profile was not considered a variable in this preliminary study.

When assessing a possible correlation between PMI and the oxidised cysteic acid (Table 5), statistically significant differences were found (*p* < 0.05) for the three degradation indices despite the small number of individuals in the study (n = 14). The obtained results show that an increase in the PMI leads to a reduction in the ratios considered. This data illustrates that the content of cysteic acid increases with time, confirming the initial hypothesis posed by the authors.

To evaluate if the previous correlations would change with burial conditions, spectroscopic data was separated by burial type. Here, statistically significant differences (*p* < 0.05) were found in vault burials for all indices. These results might reflect the long PMI of the individuals (40–131 years), implying an increase in intensity of the cysteic acid bands over time. Degradation indices I and II showed a correlation with PMI (*p* < 0.05) in soil burials, while degradation index II was the only one offering a correlation with PMI (*p* = 8.42e^−4^) in the aerobic consumption modules. In both situations, the overall PMI range (6–28 years) is not as wide as in vaults which might impact the obtained results. Furthermore, hair fibres from the individuals buried in the aerobic consumption modules might not have been intensively degraded for two main reasons: i) the keratinolytic microorganisms might have been absent or in a low population number; and ii) the microbial degradation process might have been inhibited due to the lack of a hospitable environment for microbial activity [11]. This degradation inhibition might have been concomitant with the interruption of the cadaveric decomposition given that both individuals entombed in the aerobic consumption modules were completely mummified. Even though individual CMA/40 and individual CMA/19 had been inhumed for 40 years in distinct vaults of the same cemetery, results from analysis exhibited differences in hair degradation between these two individuals.

Human hair can vary greatly in many characteristics, not only macroscopically (e.g., in colour, shape and size) but also in its microstructure [12]. As an example, the shape and number of cuticle cell layers might influence the level of resistance to external agents and consequent damage (Fig. 5). Such variation can occur in the same individual, explaining the diversity of values obtained among the four band areas analysed as demonstrated by the results of the descriptive statistics performed (Table 4). For this reason, a thorough understanding of decomposition environments and their influence on human hair degradation is fundamental (Fig. 5).

## Conclusion

This preliminary study suggests that changes in the chemical composition of human hair have the potential for time-since-death assessment, especially when analysing the ratio between amide II (∼1550 cm^−1^) and the combined disulfide monoxide symmetrical stretch (∼1074 cm^−1^) and the shouldering SO_3_ symmetric stretch (∼1043 cm^−1^) bands of cysteic acid. Here, the authors found a correlation indicating that the longer the PMI of an individual, the smaller the resultant ratio due to an increased intensity of disulfide bond (S = O) oxidation. Considering these promising results, it is encouraged that further studies should be conducted with a larger number of samples, a wider range of PMIs, and a greater variety of depositional environments.
